# Emotional and behavioral problems of 7-11 year old children in war-torn nagorno – karabakh region in azerbaijan

**DOI:** 10.1192/j.eurpsy.2021.1670

**Published:** 2021-08-13

**Authors:** N. Osmanli, A. Babayev, I. Rustamov, K. Munir

**Affiliations:** 1 Psychological Counseling, Nefes Mental Health Academy, Baku, Azerbaijan; 2 Psychology, Khazar University, Baku, Azerbaijan; 3 Psychiatry, Azerbaijan Medical University, Baku, Azerbaijan; 4 Psychiatry, Harvard Medical School, Boston, United States of America

**Keywords:** Emotional and behavioral problems, child mental health, Nagorno-Karabakh conflict, Azerbaijan

## Abstract

**Introduction:**

The present aimed to examine the mental health conditions of children, ages 7-11 years, living in conditions of war and conflict conditions in two districts of a Nagorno-Karabakh, Azerbaijan.

**Objectives:**

The study surveyed teachers of 617 primary school children (mean age 8.9, SD 1.24; 50.7% female) across nine schools in Agdam and Karabakh districts.

**Methods:**

The children were evaluated with the previously validated Azerbaijani version of the Strengths and Difficulties Questionnaire (SDQ) Teacher Form. The total difficulty and five subscale scores (emotional problems, conduct problems, hyperactivity/inattention, peer relationship problems, and prosocial behavior) were assessed.

**Results:**

About a third of children (32.7%) had abnormal total scores, and a fifth (21.4%) were in borderline range. The SDQ subscale scores included emotional problems (19.4%); conduct problems (20.3%), hyperactivity/inattention (12.2%), peer relationship problems (31.1%), and pro-social behavior difficulties (13.1%). Boys had higher level of difficulties than females (p<.01) with a negative correlation of children’s school performance with maternal education.

**Conclusions:**

The findings of the study show that more than half of the children living in the war zone in Azerbaijan have significant mental health problems. The psychological effects of the war environments have a profund effect on child development and education and need to be revisited under the United Nations Sustainable Development Goals. These include the provision of implicit supports in terms of their emotional, behavioral, psychosocial development and education of children and protection of children from wars, conflicts, and persecution.

**Disclosure:**

No significant relationships.

